# Bushen Huoxue formula protects against renal fibrosis and pyroptosis in chronic kidney disease by inhibiting ROS/NLRP3-mediated inflammasome activation

**DOI:** 10.1080/0886022X.2024.2354444

**Published:** 2024-05-24

**Authors:** Lin Liao, Pengyu Tao, Qiming Xu, Jie Chen, Weiwei Liu, Jing Hu, Jianrao Lu

**Affiliations:** Department of Nephrology, Seventh People’s Hospital Affiliated to Shanghai University of Traditional Chinese Medicine, Shanghai, China

**Keywords:** Bushen huoxue, NLRP3, pyroptosis, renal fibrosis, network pharmacology

## Abstract

**Background:**

Renal fibrosis contributes to chronic renal failure and a decline in the quality of life. Bushen Huoxue (BSHX) formula is a Traditional Chinese Medicine used to treat chronic renal failure. However, its mechanisms of action remain unclear.

**Methods and results:**

In this study, a rat model of renal fibrosis was constructed by 5/6 nephrectomy *in vivo*, and histopathological changes were analyzed using hematoxylin-eosin and Masson’s trichrome staining. Angiotensin II (Ang II) was used to establish an *in vitro* renal fibrosis cell model *in vitro*. Pyroptosis was measured using flow cytometry. Related markers of fibrosis and NOD-like receptor protein 3 (NLRP3) inflammasome activation were measured using western blotting and enzyme-linked immunosorbent assay. Treatment with BSHX (0.25, 0.5, and 1 g/kg) significantly inhibited renal fibrosis and damage in 5/6 nephrectomized rats and simultaneously reduced oxidative stress and NLRP3 inflammasome activation. Similarly, BSHX treatment reduced the levels of hydroxyproline, transforming growth factor-β, matrix metalloproteinase 2, and matrix metalloproteinase 9 and inactivated the Smad2/3 signaling pathway in Ang II-treated HK-2 cells. Our data also showed that treatment with BSHX reduced NLRP3 inflammasome activation and pyroptosis in Ang II-treated HK-2 cells. Moreover, fibrosis and pyroptosis in HK-2 cells induced by NLRP3 overexpression were reduced by treatment with BSHX.

**Conclusions:**

BSHX significantly reduced renal fibrosis and pyroptosis, and its mechanism was mainly associated with the inhibition of reactive oxygen species (ROS)/NLRP3-mediated inflammasome activation.

## 1. Introduction

Chronic kidney disease (CKD), a common disease caused by external and internal stimuli, manifests as structural renal damage and dysfunction, which is associated with increased morbidity and usually develops into renal fibrosis [1,2]. Accumulation of the extracellular matrix and tubular atrophy have been identified as the main causes of renal fibrosis [3,4]. Inflammation, epithelial-mesenchymal transition, and myofibroblast activation contribute to the pathogenesis of renal fibrosis [5,6]. However, the underlying mechanisms of renal fibrosis need to be further investigated, and potential solutions for the treatment of renal fibrosis are urgently needed [7,8]. The 5/6 nephrectomized rat model is a commonly used model of chronic renal failure that studies mechanisms and drug interventions for renal fibrosis and inflammation [9]. Previous studies have demonstrated that the intrarenal renin-angiotensin system (RAS) is activated in 5/6 nephrectomized rats [10]. The 5/6 nephrectomized rat model is also associated with increased levels of angiotensin II (Ang II) [11], which is a potent oxidative stress factor that plays a significant role in the development of renal fibrosis and inflammation [12,13].

Traditional Chinese Medicine (TCM) has a rich history of clinical application in the treatment of various chronic diseases in China. One notable TCM is Bushen Huoxue (BSHX) formula, which comprises the following five medicinal plants: *Astragalus mongholicus* (Huang Qi), *Trigonella foenum-graecum* L. (Hu Luba), *Rheum palmatum* L. (Da Huang), *Vaccaria segetalis* (Wang Buliuxing), and *Curcuma phaeocaulis* Val. (E Zhu). BSHX is widely used in clinical practice in China to treat chronic renal failure, as it functions to tonify the kidneys and promote blood circulation. In our previous study, BSHX was found to improve renal function in rats subjected to 5/6 nephrectomy [14,15]. Based on these previous findings, the present study aimed to further investigate the molecular mechanisms underlying the beneficial effects of BSHX on renal fibrosis.

Pyroptosis is a newly discovered form of programmed cell death characterized by membrane rupture, cell swelling, and caspase-1 activation [16]. Pyroptosis is regulated by the gasdermin (GSDM) family of proteins, leading to the secretion of pro-inflammatory cytokines [17]. Interleukin-1beta (IL-1β) and IL-18 are two important pro-inflammatory cytokines involved in pyroptosis. As an important upstream protein of the gasdermin D-N-terminal domain (GSDMD-N), inflammasome activation is linked to the maturation of IL-1β and IL-18 *via* the activation of caspase-1 [18]. Furthermore, pyroptosis is positively associated with fibrosis, and inhibition of inflammasome-related proteins can delay fibrosis development [19]. Caspase-1-mediated pyroptosis drives renal inflammation and fibrosis [20]. NOD-like receptor protein 3 (NLRP3)-caspase-1-GSDMD-N, the classical signaling pathway involved in pyroptosis, is mediated by several factors, including lysosomal permeabilization and reactive oxygen species (ROS) production [21,22]. In this study, we aimed to investigate the efficacy and mechanism of action of a medicine (BSHX) with antioxidative and anti-inflammatory functions, but few side effects in alleviating renal fibrosis.

## 2. Materials and methods

### 2.1. Preparation of BSHX

BSHX comprises five components, including *Astragalus mongholicus* (Huang Qi), 15 g; *Trigonella foenum-graecum* L. (Hu Luba), 15 g; *Rheum palmatum* L. (Da Huang), 15 g; *Vaccaria segetalis* (Wang Buliuxing), 15 g; *Curcuma phaeocaulis* Val. (E Zhu), 15 g. All crude drugs and drug preparations were provided by the TCM Pharmacy Department of Shuguang Hospital, which is affiliated with Shanghai University of TCM.

### 2.2. HPLC analysis

The components of BSHX were measured using an Agilent 1100 HPLC system equipped with a quaternary pump, auto-sampler, degasser, automatic thermostatic column compartment, DAD, and LC/MSD Trap XCT ESI mass spectrometer (Agilent Technologies, MA, USA), as previously described [23].

### 2.3. Animal model

Thirty male Sprague–Dawley (SD) rats weighing 150–200 g were purchased from Shanghai SLAC Laboratory Animal Co. Ltd. (Shanghai, China). Twenty-four rats underwent 5/6 nephrectomy (5/6 Nx), and six rats were assigned to the sham group. After anesthesia with isoflurane/oxygen (1:5), two-thirds of the left kidney was excised. One week later, the right kidney was excised. All rats subjected to 5/6 Nx were randomly assigned to four groups: 5/6 Nx group; 5/6 Nx + 0.25 g/kg BSHX group; 5/6 Nx + 0.5 g/kg BSHX group; 5/6 Nx + 1 g/kg BSHX group. BSHX was administered once daily by oral gavage. After 1 month of treatment, systolic blood pressure was measured using the tail-cuff method (MK-2000; Muromachi Kikai, Tokyo, Japan). All rats were sacrificed after 1 month of treatment. Creatinine, blood urea nitrogen (BUN), and urine protein levels were analyzed using the Creatinine Assay Kit (C011-1-1), Urea Assay Kit (C013-2-1), and Urine Protein Assay Kit (C035-2-1; all from Nanjing Jiancheng Bioengineering Institute, Nanjing, China), respectively, according to the manufacturer’s instructions. Renal tissues were immediately excised and fixed with 4% paraformaldehyde or snap-frozen at −80 °C. The protocols were reviewed and approved by the Animal Care Committee of the Seventh People’s Hospital affiliated with Shanghai University of Traditional Chinese Medicine (PZSHUTCM220711030).

### 2.4. Histopathology assay

After fixing in 10% formalin for 24 h, renal tissues were embedded in paraffin, cut into slices, deparaffinized with xylene baths, and rehydrated with graded alcohols. Hematoxylin and eosin (H&E) staining was performed as follows: (1) hematoxylin staining for 5 min at 37 °C and (2) 0.5% eosin for 2 min. Masson’s trichrome staining was performed according to the manufacturer’s protocol (Leagene, Beijing, China). Myofibers are shown in red, while collagen fibers are indicated by light green or aniline blue staining. Images were captured using an optical microscope (Leica).

### 2.5. Cell culture

Human renal proximal tubular epithelial HK-2 cells (Cell Bank of Shanghai Biology Institute, China) were cultured in Dulbecco’s modified Eagle’s medium (DMEM) containing 10% fetal bovine serum (Gibco, USA) and 1% penicillin/streptomycin (Gibco) in an incubator (37 °C, 95%, CO_2_:5%). The cells in the vehicle group were cultured in DMEM + vehicle. Cells in the Ang II group were cultured in DMEM with +1 µM Ang II. Cells in the N-acetylcysteine (NAC) group were cultured in DMEM + 1 µM Ang II + 100 µM NAC (Selleck, Radnor, PA, USA), which was used as a positive control. The cells in the BSHX group were cultured in DMEM +1 µM Ang II + BSHX (0.1, 0.2, or 0.5 mg/mL). Finally, cells were collected for further assays after 24 h of treatment.

### 2.6. Cell counting kit-8 assay

Cell viability was determined using a CCK-8 kit (CK04; Dojindo Molecular Technologies, Kumamoto, Japan). Briefly, cells were cultured in a 96-well plate (3 × 10^3^ cells per well). Then, 10 μL CCK-8 solution was added to each well, and the reaction time was maintained for 1 h. The absorbance value was obtained by reading the plate using a microplate reader (Pulangxin, Beijing, China) at 450 nm.

### 2.7. Overexpression of NLRP3

A lentivirus targeting NLRP3 was used to overexpress NLRP3 and was purchased from Genechem (Shanghai, China).

### 2.8. Biochemical determination

The activities of malondialdehyde (MDA), glutathione (GSH), superoxide dismutase (SOD), and hydroxyproline (Hyp) were each analyzed using commercial kits (Nanjing Jiancheng Bioengineering Institute, Nanjing, China).

### 2.9. An enzyme-linked immunosorbent assay (ELISA)

The concentrations of transforming growth factor-β (TGF-β), matrix metalloproteinase 2 (MMP2), MMP9, IL-1β, and IL-18 in HK-2 cell supernatant and serum were measured using ELISA kits (CUSABIO, Houston, TX, USA). The OD450 value was measured using a microplate reader (Bio-Rad Laboratories Inc., Hercules, CA, USA).

### 2.10. Measurement of intracellular ROS production

After treatment with Ang II and BSHX, HK-2 cells were incubated in phosphate buffer saline containing 20 µM 2′,7′-dichlorodihydrofluorescein diacetate (DCFH-DA) at 37 °C for 20 min. Subsequently, the cells were washed with phosphate buffer saline and harvested into tubes. Intracellular ROS production was measured by flow cytometry (BD Biosciences, San Jose, CA, USA).

### 2.11. Cell pyroptosis

Propidium iodide (PI) and caspase-1 p20 staining were used to determine pyroptotic cell death. Cells (5 × 105 cells/well) were cultured in a six-well plate, reached 50% confluence and were then conditioned with caspase-1 p20 antibody/FITC (Eterlife Ltd., UK) and 10 μl PI (Thermo Fisher) for 15 min. Flow cytometry was used to analyze the samples.

### 2.12. Western blotting

Western blotting was performed according to the manufacturer’s instructions. Radioimmunoprecipitation assay lysis buffer and EDTA-free protease inhibitor cocktail (Roche, Germany) were used for protein extraction from kidney and cell samples, and the protein concentration was determined using the BCA protein assay. After blocking with 5% skim milk, the membranes were incubated with primary antibodies, secondary antibodies, and electrochemiluminescence reagents (Bio-Rad Laboratories, Inc.). Protein levels were analyzed using Quantity One software (Bio-Rad Laboratories, Inc.), with glyceraldehyde 3-phosphate dehydrogenase (GAPDH) as the loading control. The following primary antibodies were used: NLRP3 (DF7438, Affinity, USA), Caspase-1 p20 (AF4005, Affinity, USA), GSDMD-N (ab215203, Abcam, USA), TGF-β (AF1027, Affinity, USA), Smad2/3 (AF6367, Affinity), p-Smad2/3 (AF3367, Affinity, USA), and GAPDH (60004-1-Ig, Proteintech, USA).

### 2.13. Network pharmacology

The components of BSHX were obtained from the Traditional Chinese Medicine Systems Pharmacology (TCMSP) database. Targets were searched in GeneCards using the keywords “renal fibrosis, chronic kidney disease”. A Venn diagram was drawn to identify the overlapping genes of BSHX and renal fibrosis. The Database for Annotation, Visualization, and Integrated Discovery (DAVID) platform was used for the Kyoto Encyclopedia of Genes and Genomes (KEGG) enrichment analyses.

### 2.14. Statistical analysis

All data are presented as the mean ± standard deviation (SD). All experiments were performed at least three times. One-way analysis of variance (ANOVA) followed by Dunnett’s test for multiple comparisons used, and *p* < 0.05 indicates statistical significance.

## 3. Results

### 3.1. Results of high‑performance liquid chromatography coupled with electrospray mass spectrometry (HPLC/ESI–MS) assays for BSHX

**Figure 1.**
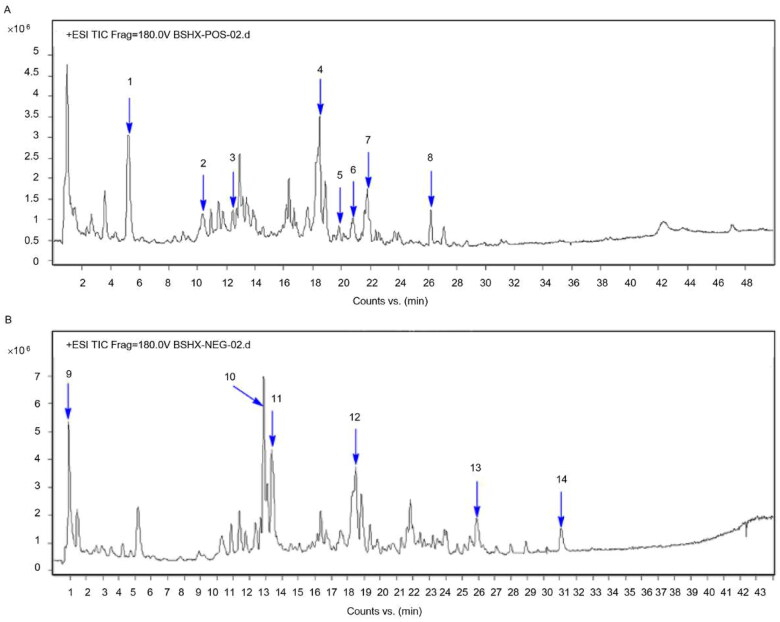
HPLC-ESI–MS chromatogram of the BSHX extract in (A) positive and (B) negative mode.

The aqueous extracts of BSHX were analyzed by HPLC/ESI–MS in positive and negative ion mode (Figure 1). Fourteen compounds were identified: astragaloside (1), vaccarin (2), emodin-8-β-D-glucoside (3), sennoside A (4), vaccarin (5), isocurcumenol (6), trigonelline (7), sennoside E (8), gallocatechin (9), carpaine (10), aloe-emodin (11), chrysophanol (12), rhein (13), and emodin (14).

### 3.2. BSHX improves renal function and fibrosis in 5/6 Nx rats

**Figure 2.**
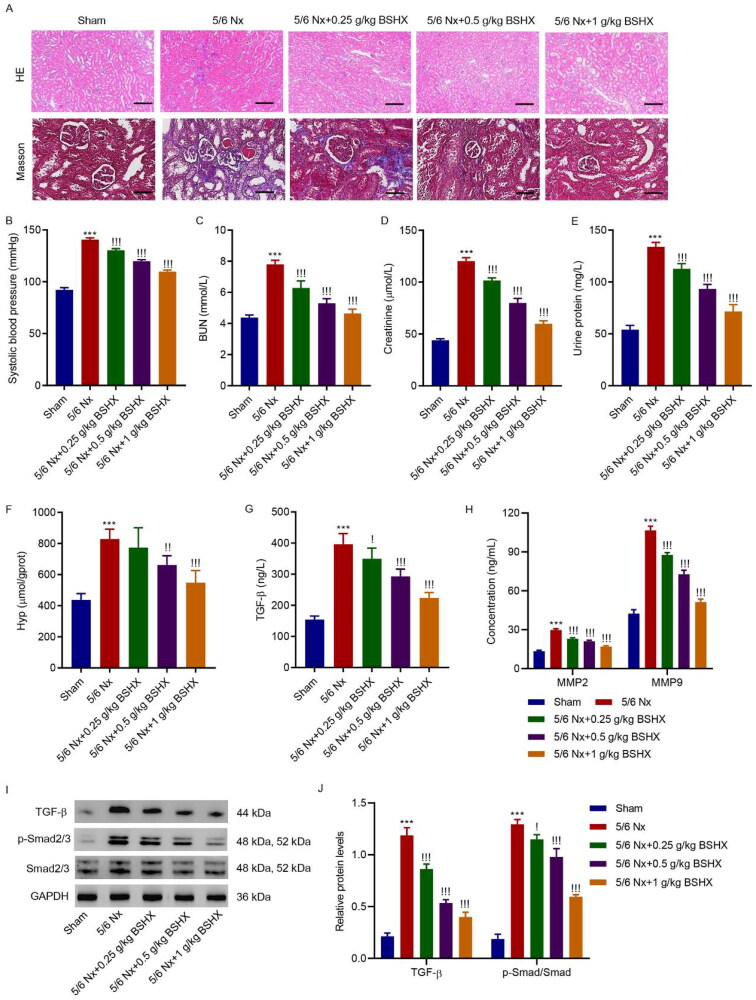
BSHX improves renal function and fibrosis in 5/6 Nx rats. (A) HE (Scale bar, 200 µm. Magnification ×100) and Masson staining (Scale bar, 100 µm. Magnification ×200). The (B) systolic blood pressure, levels of (C) BUN, (D) creatinine, (E) urinary protein, (F) Hyp, (G) TGF-β, (H) MMP2 and MMP9 and (I, J) expression of TGF-β, p-Smad2/3 and Smad2/3. Data represent the mean ± SD (*n* = 3 or 6). One-way ANOVA followed by Dunnett’s test for multiple comparisons was used. ***p < 0.001 vs. Sham group. !*p* < 0.05, !!*p* < 0.01, !!!*p* < 0.001 vs. the 5/6 Nx group. HE, hematoxylin and eosin; BUN, blood urea nitrogen; Hpy, hydroxyproline; TGF-β, transforming growth factor-beta; MMP2, matrix metalloproteinase 2; MMP9: matrix metalloproteinase 9; GAPDH, glyceraldehyde 3-phosphate dehydrogenase.

To investigate the functional role of BSHX in regulating renal fibrosis, we used 5/6 Nx SD rats to construct a renal fibrosis model and administered different concentrations of BSHX. H&E and Masson staining showed that 5/6 nephrectomy resulted in severe kidney structural impairment and collagen deposition in rats, which were ameliorated by treatment with BSHX (Figure 2(A)). 5/6 Nx significantly increased systolic blood pressure, and BSHX treatment significantly inhibited the increase in systolic blood pressure induced by 5/6 Nx (Figure 2(B)). Moreover, renal function indices, including BUN, creatinine, and urinary protein, were significantly increased in 5/6 Nx rats, which was reversed by BSHX treatment (Figure 2(C–E)). As shown in Figure 2(F–H), 5/6 Nx contributed to increased Hyp levels in renal tissues and serum levels of TGF-β, MMP2, and MMP9, which were improved by treatment with BSHX. To further identify the related signaling pathways that mediate the interaction between BSHX and CKD, we performed a KEGG enrichment analysis of the common targets of BSHX and CKD. The top 10 signaling pathways for KEGG enrichment analysis are shown in Fig S1. Similarly, 5/6 Nx contributed to the activation of the TGF-β/Smad2/3 signaling pathway in renal tissues, which was improved by treatment with BSHX (Figure 2(I–J)).

### 3.3. BSHX attenuates oxidative stress and NLRP3 inflammasome activation in 5/6 Nx rats

**Figure 3.**
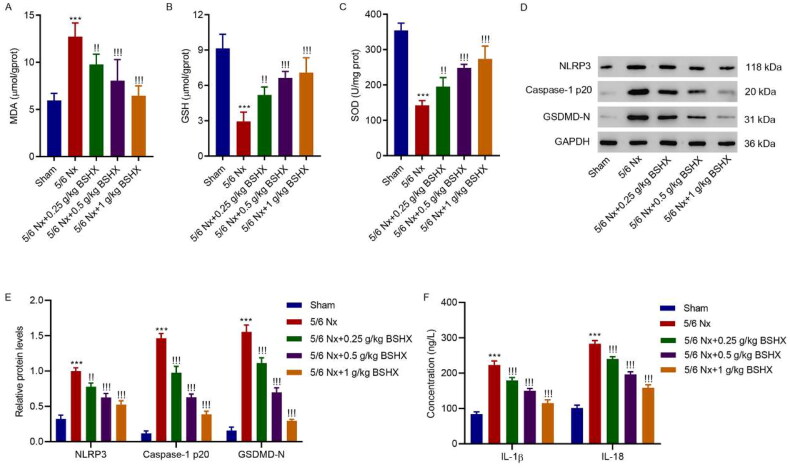
BSHX attenuates oxidative stress and NLRP3 inflammasome activation in 5/6 Nx rats. The activities of (A) MDA, (B) GSH and (C) SOD, (D, E) expression of NLRP3, caspase-1 p20, GSDMD-N, and (F) release of IL-1β and IL-18. Data represent the mean ± SD (*n* = 3 or 6). One-way ANOVA followed by Dunnett’s test for multiple comparisons was used. ***p < 0.001 vs. Sham group. !*p* < 0.05, !!*p* < 0.01, !!!*p* < 0.001 vs. the 5/6 Nx group. MDA, malondialdehyde; GSH, glutathione; SOD, superoxide dismutase; NLRP3, NOD-like receptor protein 3; GSDMD-N, gasdermin D-N-terminal domain; IL-1β, interleukin-1beta; IL-18, interleukin-18; GAPDH, glyceraldehyde 3-phosphate dehydrogenase.

The oxidative stress levels were measured. Rats with 5/6 Nx exhibited higher activity of MDA and lower activities of SOD and GSH than rats with sham surgery, which was corrected by treatment with BSHX (Figure 3(A–C)). As reported previously [19], NLRP3 inflammasome activation is associated with fibrosis, and we aimed to clarify whether an interaction exists between BSHX and NLRP3 inflammasome activation. The results shown in Figure 3(D–F) showed that NLRP3, caspase-1 p20, GSDMD-N, IL-1β, and IL-18 protein levels increased, which decreased after BSHX administration.

### 3.4. BSHX alleviates Ang II-induced fibrosis and oxidative stress in HK-2 cells

**Figure 4.**
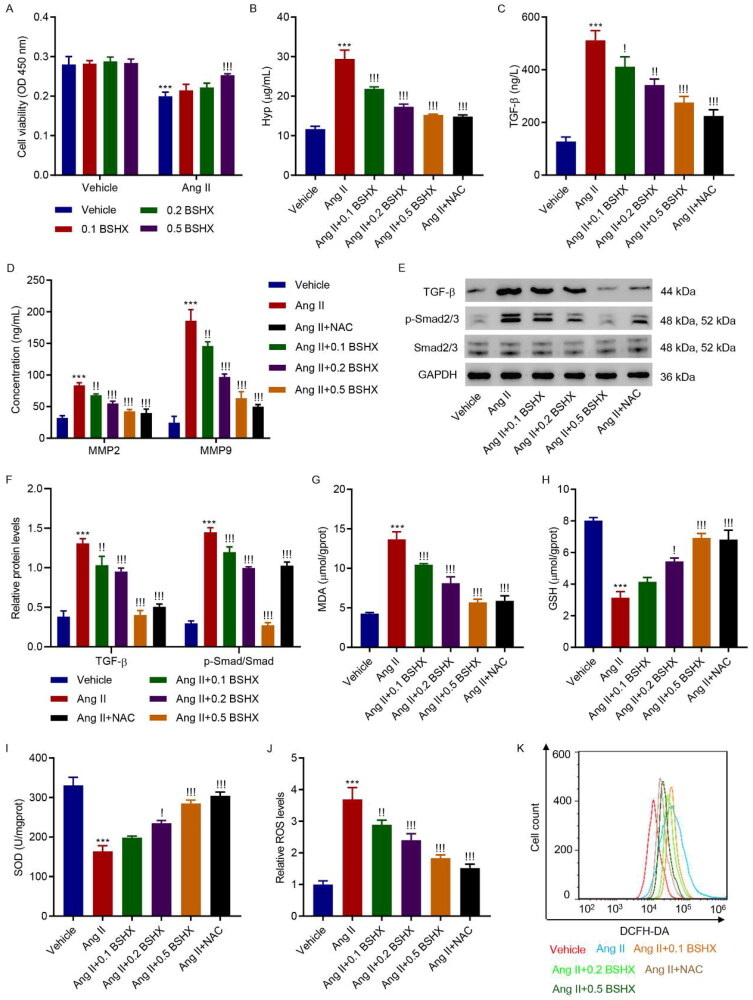
BSHX alleviates Ang II-induced fibrosis and oxidative stress in HK-2 cells. (A) Cell viability in HK-2 cells treated with Ang II (1 µM) in the absence or presence of different concentrations of BSHX (0.1, 0.2, and 0.5 mg/mL). The (B) Hyp, (C) TGF-β, (D) MMP2 and MMP9 levels, (E, F) expression of TGF-β, p-Smad2/3 and Smad2/3, activities of (G) MDA, (H) GSH, (I) SOD, and (J, K) ROS production were detected in HK-2 cells treated with Ang II (1 µM), different concentrations of BSHX (0.1, 0.2, and 0.5 mg/mL), and 100 µM NAC as positive control. Data represent the mean ± SD (*n* = 3). One-way ANOVA followed by Dunnett’s multiple comparisons test was used. ***p < 0.001 vs. Vehicle group. !*p* < 0.05, !!*p* < 0.01, !!!*p* < 0.001 vs. the Ang II group. Ang II, angiotensin II; Hpy, hydroxyproline; TGF-β, transforming growth factor-beta; MMP2, matrix metalloproteinase 2; MMP9: matrix metalloproteinase 9; MDA, malondialdehyde; GSH, glutathione; SOD, superoxide dismutase; ROS, reactive oxygen species; DCFH-DA, 2’,7'-dichlorodihydrofluorescein diacetate; NAC, N-acetylcysteine; GAPDH, glyceraldehyde 3-phosphate dehydrogenase.

To further investigate the role of BSHX in fibrosis and oxidative stress, HK-2 cells were treated with Ang II (1 µM) and different concentrations of BSHX (0.1, 0.2, and 0.5 mg/mL). The CCK-8 assay showed that treatment with 0.5 mg/mL BSHX significantly inhibited the Ang II-induced decrease in cell viability (Figure 4(A)). As shown in Figure 4(B–F), Ang II significantly increased the levels of Hyp, TGF-β, MMP2, and MMP9, along with significant activation of the Smad2/3 signaling pathway, which was inhibited by BSHX treatment. In addition, Ang II increased MDA and ROS levels and decreased antioxidant enzyme activities (GSH and SOD), which were reversed by BSHX treatment (Figure 4(G–K)).

### 3.5. BSHX alleviates Ang II-induced NLRP3 inflammasome activation and pyroptosis in HK-2 cells

**Figure 5.**
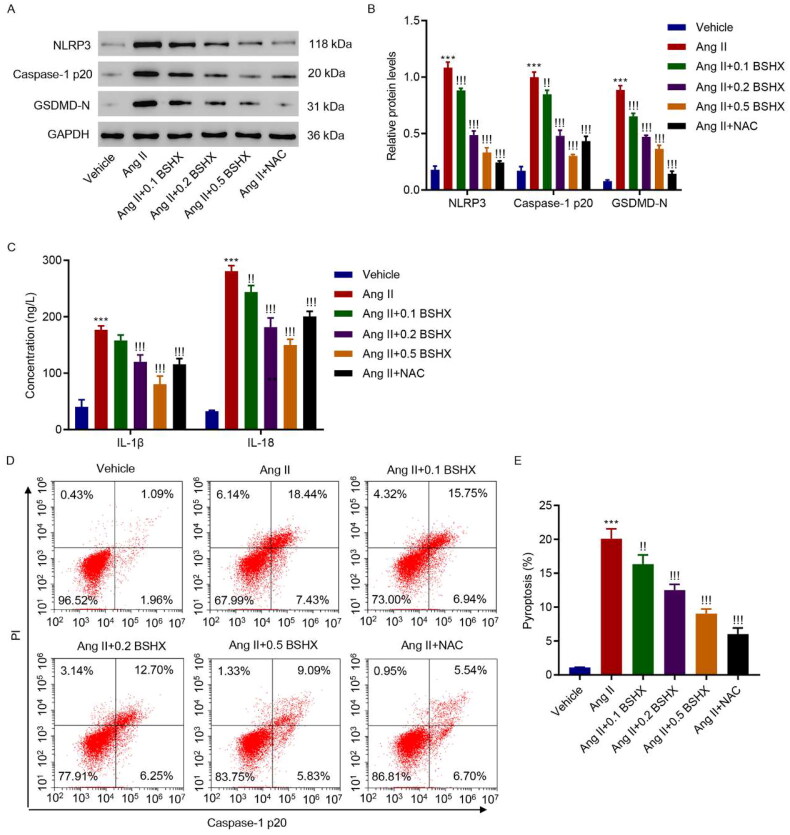
BSHX alleviates Ang II-induced NLRP3 inflammasome activation and pyroptosis in HK-2 cells. (A, B) The protein levels of NLRP3, caspase-1 p20, and GSDMD-N, (C) concentration of IL-1β and IL-18, and (D, E) cell pyroptosis were detected in HK-2 cells treated with Ang II (1 µM), different concentrations of BSHX (0.1, 0.2, and 0.5 mg/mL), and 100 µM NAC as positive control. Data represent the mean ± SD (*n* = 3). One-way ANOVA followed by Dunnett’s test for multiple comparisons was used. ***p < 0.001 vs. Vehicle group. !*p* < 0.05, !!*p* < 0.01, !!!*p* < 0.001 vs. the Ang II group. NLRP3, NOD-like receptor protein 3; GSDMD-N, gasdermin D-N-terminal domain; IL-1β, interleukin-1beta; IL-18, interleukin-18; PI, propidium iodide; NAC, N-acetylcysteine; GAPDH, glyceraldehyde 3-phosphate dehydrogenase.

Next, markers related to NLRP3 inflammasome activation were detected using western blotting and ELISA. The results shown in Figure 5(A–C) indicate that the levels of NLRP3, caspase-1 p20, GSDMD-N, IL-1β, and IL-18 were upregulated in the Ang II group, whereas BSHX administration reduced the levels of these pyroptosis markers. NLRP3-caspase-1-GSDMD is a classic signaling pathway involved in pyroptosis [21]. Therefore, pyroptosis was measured using flow cytometry. As shown in Figure 5(D,E), BSHX treatment alleviated Ang II-induced increase in pyroptosis in HK-2 cells.

### 3.6. BSHX suppresses the NLRP3-mediated fibrosis and pyroptosis in HK-2 cells

**Figure 6.**
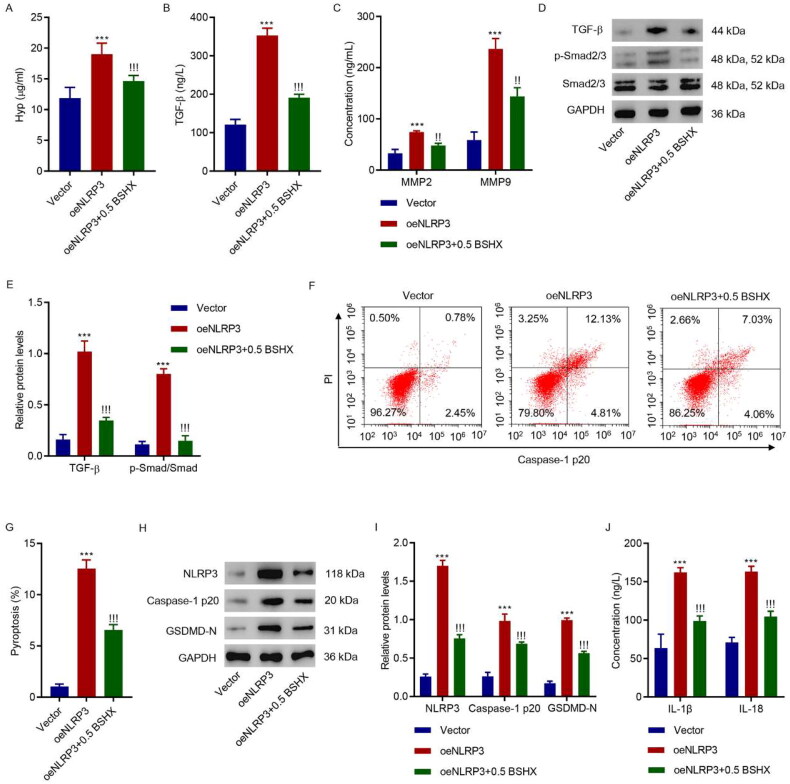
BSHX suppresses NLRP3-mediated fibrosis and pyroptosis in HK-2 cells. (A) Hyp, (B) TGF-β, (C) MMP2 and MMP9 levels, (D, E) expression of TGF-β, p-Smad2/3, and Smad2/3, (F, G) cell pyroptosis, (H, I) the protein levels of NLRP3, caspase-1 p20, and GSDMD-N, and (J) concentration of IL-1β and IL-18 in HK-2 cells transduced with NLRP3 overexpressing lentivirus in the presence of 0.5 mg/mL BSHX. Data represent the mean ± SD (*n* = 3). One-way ANOVA followed by Dunnett’s test for multiple comparisons was used. ***p < 0.001 vs. Vector group. !!!*p* < 0.001 vs. the oeNLRP3 group. Hpy, hydroxyproline; TGF-β, transforming growth factor-beta; MMP2, matrix metalloproteinase 2; MMP9, matrix metalloproteinase 9; NLRP3, NOD-like receptor protein 3; GSDMD-N, gasdermin D-N-terminal domain; IL-1β, interleukin-1beta; IL-18, interleukin-18; PI, propidium iodide; GAPDH, glyceraldehyde 3-phosphate dehydrogenase.

We further investigated the role of NLRP3 in the effects of BSHX on fibrosis and pyroptosis in HK-2 cells. Overexpressing lentivirus targeting NLRP3 was applied to HK-2 cells together with 0.5 mg/mL BSHX. As shown in Figure 6(A–E), NLRP3 overexpression increased the levels of Hyp, TGF-β, MMP2, and MMP9 and activated the Smad2/3 signaling pathway; however, these effects were alleviated by BSHX treatment in HK-2 cells. Furthermore, BSHX treatment alleviated the increase in pyroptosis induced by NLRP3 overexpression (Figure 6(F,G)). Consistent with this, NLRP3 overexpression increased the protein levels of NLRP3, caspase-1 p20, GSDMD-N, IL-1β, and IL-18, and these effects were alleviated by BSHX treatment (Figure 6(H–J)). Overall, BSHX regulation of fibrosis and pyroptosis in HK-2 cells is mediated by NLRP3.

## 4. Discussion

The 5/6 Nx rat model is a commonly used model of chronic renal failure that studies mechanisms and drug interven­tions for renal fibrosis and inflammation [9]. Our previous studies showed that BSHX protects against renal fibrosis and improves renal function in rats with 5/6 Nx by regulating the expression of TNF-𝛼, NF-𝜅B, E-cadherin, α-SMA, TGF-β1, CTGF, PPAR𝛾, OPN, fibronectin, and laminins [14,15], suggesting an important role of BSHX in alleviating renal fibrosis. Previous studies have demonstrated that the intrarenal RAS is activated in 5/6 Nx rats [10], and the 5/6 Nx rat model is also associated with increased levels of Ang II [11], which is a potent renal growth factor that plays a significant role in the development of fibrosis. The mechanism of Ang II involvement in renal fibrosis is associated with the stimulation of the release of pro-inflammatory and pro-fibrogenic cytokines, which contribute to the accumulation of extracellular matrix and subsequent fibrotic processes [24]. Our results suggest that BSHX plays an antioxidant role in combating Ang II-induced oxidative stress and potentially contributes to its protective effects against fibrosis development, further supporting the potential therapeutic value of BSHX in the treatment of renal fibrosis and highlighting its ability to modulate antioxidant mechanisms in renal cells.

Matrix metalloproteinases (MMPs) possess many biological functions, such as tissue remodeling and growth, wound repair, defense mechanisms, and immune responses [25]. MMPs can be categorized as collagenases, gelatinases, stromelysins, matrilysins, and membrane-type MMPs. MMP2 and MMP9 are members of the gelatinase subfamily [26]. Several studies have shown that ROS accumulation promotes the expression of MMP2, and MMP9 participates in all stages of different forms of CKD, which is characterized by renal fibrosis [25,27]. Consistent with this, increases in both MMP2 and MMP9 were observed in Ang II-treated HK-2 cells, which could be alleviated by treatment with BSHX. Kidney injury is often replaced by renal scarring, which later transforms into renal fibrosis. During the development of renal fibrosis, TGF-β serves as a key regulator of this process by inducing excessive formation of the extracellular matrix. The TGFβ1/SMAD/PAI-1 axis induces renal fibrosis [28]. Consistently, Ang II-induced activation of the TGFβ1/Smad pathway was attenuated by BSHX therapy.

In the present study, treatment with BSHX reduced pyroptosis induced by Ang II in a dose-dependent manner, suggesting that BSHX treatment has the potential to alleviate renal fibrosis, possibly by mitigating the occurrence of pyroptosis. Pyroptosis is a recently discovered form of programmed cell death characterized by inflammasome activation and differs from apoptosis and autophagy in terms of cell morphology and function [29]. A notable feature of pyroptosis is the release of inflammatory factors, including IL-1β and IL-18. The pathways involved in pyroptosis can be categorized into canonical and non-canonical pathways, which are regulated by caspase-1 and caspase-4/5/11, respectively [30,31]. Activation of the caspase-1-mediated canonical pathway leads to upregulated levels of NLRP3 and caspase-1, as well as the release of IL-1β and IL-18 inflammatory bodies [32,33]. Notably, the depletion of NLRP3 significantly alleviated the severity of renal fibrosis in mouse models [34,35]. Additionally, studies have reported that pyroptosis in renal tubules, mediated by the TNFα/Casp3/GSDME signaling pathway, leads to tubular loss and renal fibrosis [7,33]. Our results shed light on the therapeutic value of BSHX in managing renal fibrosis and highlight its potential as a modulator of pyroptotic pathways in kidneys.

Although this study provides an important insight into the mechanism of BSHX in attenuating renal fibrosis, the limitations of this study still exist. We used a crude drug combination to observe its efficacy in improving renal fibrosis. The BSHX extract should be analyzed in future studies. Second, a gene-knockout animal would be a better option to verify the effect of BSHX in treating renal fibrosis. Furthermore, transcriptomics would help us to better understand the mechanisms of action of Chinese herbal medicine. These issues will be investigated in future studies.

The evidence presented in this study strongly supports the therapeutic effect of BSHX in inhibiting renal fibrosis in both animal and cellular models. Further studies involving human patients are necessary to investigate the potential clinical application of BSHX. These studies will improve our understanding of the efficacy of BSHX as a clinical treatment for renal fibrosis.

## Supplementary Material

Supplemental Material

## Data Availability

The datasets used and/or analyzed during the current study are available from the corresponding author upon reasonable request.
