# Supplementary figures and images for: Bushen Huoxue formula protects against renal fibrosis and pyroptosis in chronic kidney disease by inhibiting ROS/NLRP3-mediated inflammasome activation
Source: Ren Fail. 2024 May 24;46(1):2354444. doi: 10.1080/0886022X.2024.2354444 (PMC11132749; doi:10.1080/0886022X.2024.2354444)

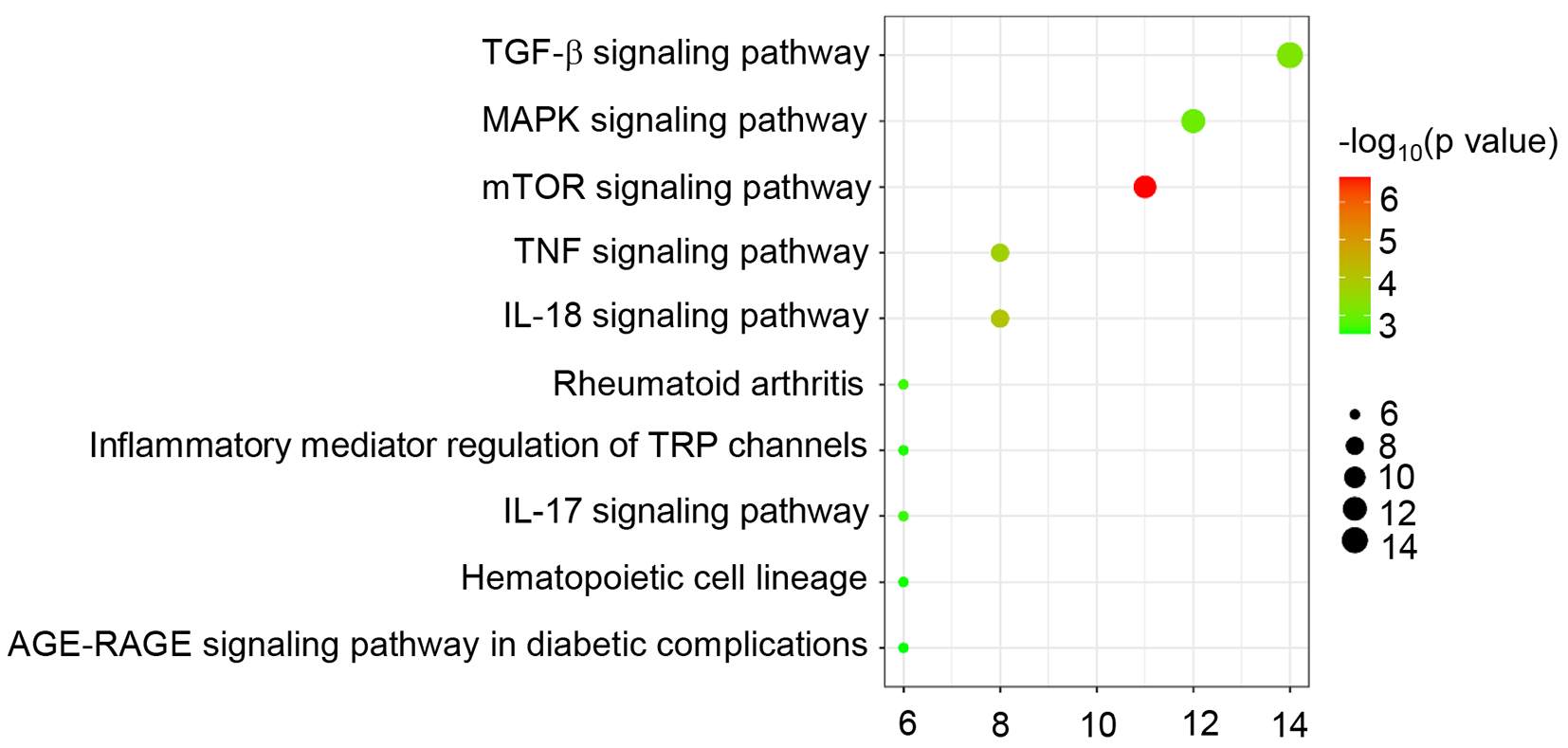

Supplement: Supplemental Material [file IRNF_A_2354444_SM5061.tif]
